# Accumulating crop functional trait data with citizen science

**DOI:** 10.1038/s41598-019-51927-x

**Published:** 2019-10-31

**Authors:** Marney E. Isaac, Adam R. Martin

**Affiliations:** 1 Department of Physical and Environmental Sciences, University of Toronto Scarborough, Toronto, Canada; 2 Department of Geography, University of Toronto, Toronto, Canada

**Keywords:** Agroecology, Sustainability

## Abstract

Trait-based ecology is greatly informed by large datasets for the analyses of inter- and intraspecific trait variation (ITV) in plants. This is especially true in trait-based agricultural research where crop ITV is high, yet crop trait data remains limited. Based on farmer-led collections, we developed and evaluated the first citizen science plant trait initiative. Here we generated a dataset of eight leaf traits for a commercially important crop species (*Daucus carota*), sampled from two distinct regions in Canada, which is 25-fold larger than datasets available in existing trait databases. Citizen-collected trait data supported analyses addressing theoretical and applied questions related to (i) intraspecific trait dimensionality, (ii) the extent and drivers of ITV, and (iii) the sampling intensity needed to derive accurate trait values. Citizen science is a viable means to enhance functional trait data coverage across terrestrial ecosystems, and in doing so, can directly support theoretical and applied trait-based analyses of plants.

## Introduction

At largest-scale of integration, analyses of trait variation among plant species have tangibly advanced our capacity to describe, predict, and manage linkages between plant diversity and ecosystem functioning^1^. At the same time, trait-based research has afforded a more nuanced assessment of how variation in the characteristics of individual plants within a species – now widely referred to as intraspecific trait variability (ITV) – influence ecosystem processes at finer scales^2,3^. Very recently the application of a functional trait-based approach to agroecological research, where ITV represents an especially large proportion of total plant trait variation in an ecosystem, has gained considerable attention^4–7^, with specific functional traits correlated to key agroecosystems processes, namely leaf morphological and chemical traits that are strongly coupled with crop resource acquisition strategies^4,5^.

Research has shown that quantifying the breadth of ITV in crops has critical implications for better understanding multiple aspects of agroecosystem structure and function including: (i) the environmental impacts of artificial selection e.g.^8^; (ii) how the functional biology of crops differs from “wild” plant species e.g.^9,10^; (iii) farm- or landscape-scale biogeochemical cycling e.g.^11^; (iv) crop yield e.g.^12–14^; and (v) crop responses to climate change e.g.^15^. Based on this growing literature, it is apparent that crop functional trait data – particularly datasets that comprehensively quantify ITV within a given crop species or genotype – greatly inform our applied and theoretical understanding of plant functional biology.

Yet while a few meta-analyses do exist^15,16^, crop trait data has largely been missing from large functional trait databases^17^. Therefore to date, crop ITV collection and analyses have largely depended on “new” samples derived from common garden experiments e.g.^8,18^ or field-based observational studies e.g.^19–21^. Although these studies have been instructive, larger and robust trait data collection initiatives are commonly pointed to being a main limitation to additional trait-based agroecological research. For example, larger and geographically broad trait data collection initiatives are critical for predicting crop production and food security under local and global environmental change^6^.

One possible avenue for enhancing our understanding of crop traits is through citizen science initiatives. The agricultural community has always maintained strong ties to the research community^22^, and citizen science initiatives (among other examples) have greatly informed plant breeding programs^23,24^ and soil amendment management treatments^25^, with recent work assessing the accuracy of farmer-generated data and developing approaches to ensure robust datasets^26^. However to date, citizen science initiatives have not been employed in any studies of functional trait ecology. While there has been some success in engaging citizen science to amass herbarium specimens^27^ or in plant phenological monitoring^28^, to our knowledge there remain no efforts to merge farmer research participation with trait data collections led by ecologists.

Here we developed and evaluated a novel citizen science approach to functional trait data collection. We specifically sought to: (i) launch a citizen science protocol across Canada focused on famer-led plant trait sample collection. We then sought to use these samples - which in our study are from five varietes of carrot (*Daucus carota* L.) - to (ii) consolidate morphological and chemical leaf functional traits among a range of crop varieties and farms, and in doing so, (iii) demonstrate how citizen-led trait collections can be used to inform empirical assessments of the drivers of ITV within crops. Finally, we sought to (iv) critically evaluate the efficacy of crop trait collections by determining the optimal number of farms and observations needed to accurately estimate functional trait values for, and the trait space occupied by, crop species.

## Results

Consistent with existing literature on crop traits^9,14^, leaf chemical traits including leaf carbon (C) and nitrogen (N) were least variable across our dataset (CV = 4.3–17.3), in comparison to leaf morphological traits including leaf area and leaf dry mass which were most variable among samples (CV = 59.7–69.6); specific leaf area (SLA), and petiole length and diameter were intermediary in terms of overall trait variability (CV = 27.0–34.2; Table S1).

**Figure 1:**
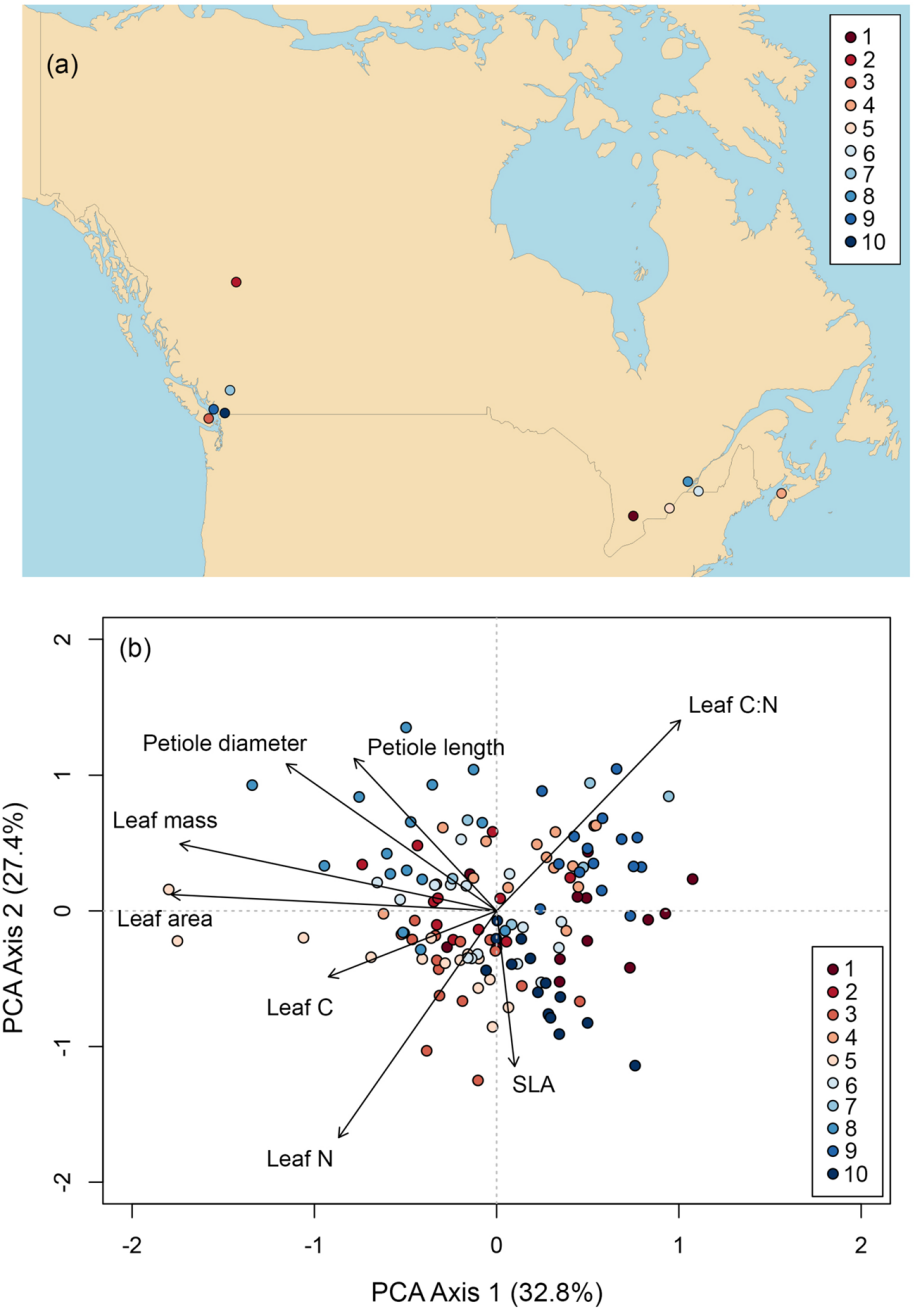
Locations of the 10 farms participating in the citizen science trait collection program in Canada (Panel a), and multivariate variation in eight leaf functional traits across these farms for five *D*. *carota* varieties (Panel b). Multivariate trait syndromes in this Principal Components Analysis (PCA) were most strongly explained by farm identity (*p* < 0.001, *r*^2^ = 0.295), therefore individual observations of leaf traits (n = 136 total) are coloured according to farm in the PCA biplot.

Across our dataset carrot leaves differentiated across two primary and significant Principal Components Analysis (PCA) axes that accounted for 32.8 and 27.4% of the variation in traits, respectively (Fig. 1b; Table S2). The first PCA axis reflected constraints associated with Corner’s Rules, such that the axis was strongly and positively related to variation in petiole diameter and length, and leaf mass and area (Fig. 1b). The second PCA reflected variation along an intraspecific Leaf Economics Spectrum (LES), being negatively associated with both SLA and leaf N, and positively associated with leaf C:N (Fig. 1b). Variability of carrot leaves in multivariate trait space was significantly related to the farm that the leaf was derived (permutational multivariate analysis of variance (PerMANOVA) *r*^2^ = 0.295, *p* < 0.001), but did not differ systematically across varieties (PerMANOVA *r*^2^ = 0.033, *p* = 0.196; Table S3). The effect of farm location on multivariate trait values did not depend on the carrot variety identity (PerMANOVA for farm-by-variety interaction term *r*^2^ = 0.137, *p* = 0.924).

**Figure 2:**
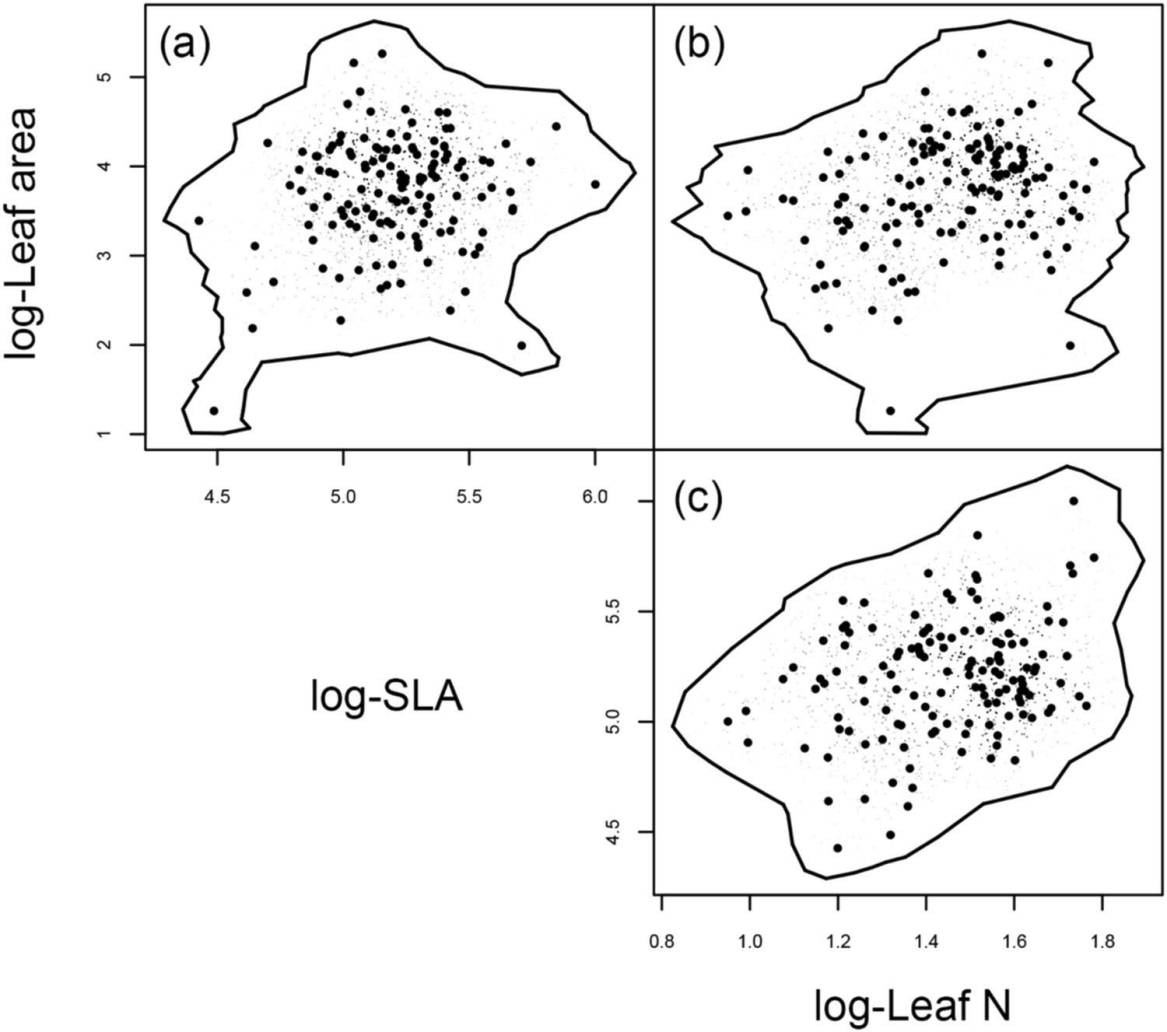
Hypervolumes for multiple bivariate functional trait axes in *D*. *carota* quantified through a citizen science trait collection program. Larger black points correspond to observations in bivariate trait space, while the solid black lines represent 2-dimensional hypervolume trait space estimated as the region capturing 95% of randomized points (small points in gray scale).

**Figure 3:**
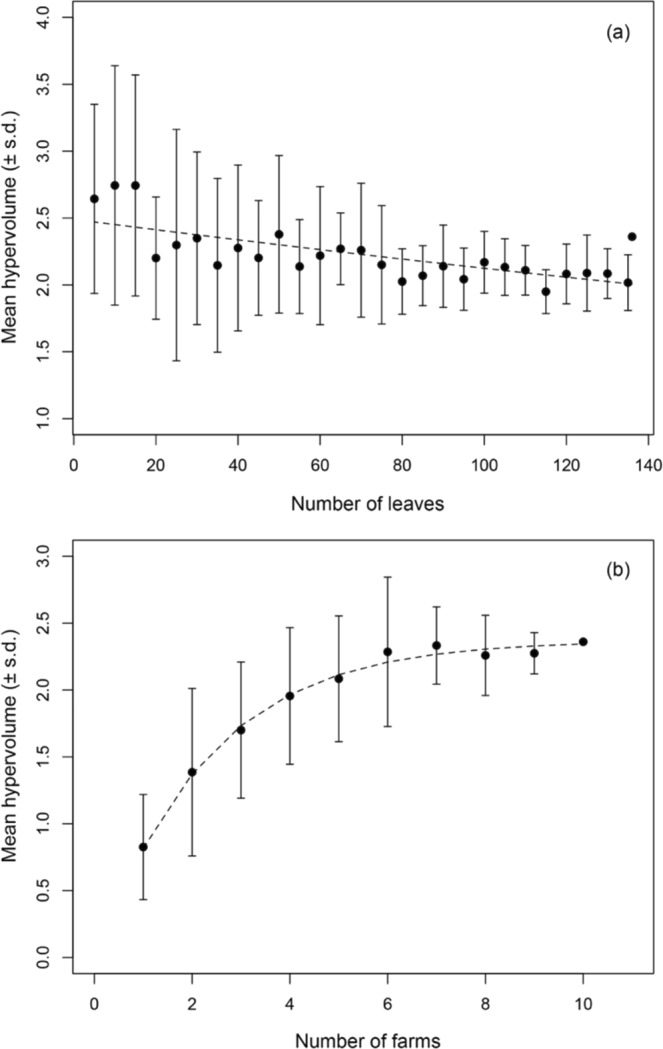
Randomization tests evaluating variation in estimated 3-dimensional trait hypervolumes in *D*. *carota* as a function of the number of leaves (Panel a) and number of farms sampled (Panel b). In both panels the right-most data point corresponds to observed hypervolumes, where *n* = 136 leaves and *n* = 10 farms (Panels a and b, respectively). All other data points represent the mean hypervolume calculated for 100 randomized datasets that included *n* = 5, 10, 15…135 leaves (Panel a) and *n* = 1,2,3…9 farms (Panel b). Error bars correspond to ±1 standard deviation, and dashed trend lines correspond to non-linear least square models fit through maximum likelihood.

Across our entire dataset, observed trait hypervolumes (*H*_*obs*_) was estimated as 2.4 with centroid values for log-transformed leaf area, leaf N, and SLA of 3.6, 1.4, and 5.3, respectively (Fig. 2). Our randomization procedures suggested that as compared to *H*_*obs*_, average randomized trait hypervolume (*H*_*rand(l)*_) values are likely to be overestimated at smaller sample sizes of individual leaves (Fig. 3a). This is particularly true where sample sizes were <20 leaves, where mean *H*_*rand(l)*_ ranged from 2.6–2.7; the largest differences were primarily associated with differences in the mean leaf area centroid calculated for the observed vs. randomized datasets (Table S4). After the threshold of 20 leaves was reached or exceeded, average *H*_*rand(l)*_ ranged between 2.0–2.4 with s.d. values that become progressively smaller (i.e. where s.d. surrounding *H*_*rand(l)*_ = 0.5 at *n*_*l*_ = 20, to a s.d. of 0.2 where *n*_*l*_ = 135), and largely encompass the observed hypervolume value (Fig. 3a, Table S4).

Changes in number of sampled farms had a much stronger and direct effect on estimated hypervolumes (Fig. 3b). Sampling only 1–3 farms reduced the hypervolume space occupied by *D*. *carota* leaves considerably where mean *H*_*rand(f)*_ ranged from 0.8–1.7 (Fig. 3b, Table S5). This again was primarily associated with differences in mean leaf area centroid values calculated in the entire dataset vs. randomized datasets (Table S5). However, when *n*_*f*_ ≥ 4, overall *H*_*rand(f)*_ values and associated s.d. values closely approximated *H*_*obs*_. Estimated s.d. values surrounding *H*_*rand(f)*_ values are notably lower where *n*_*f*_ ≤ 7 where the range of s.d. is 0.15–0.3, as compared to where *n*_*f*_ ≥ 6 where s.d. surrounding *H*_*rand(f)*_ range between 0.39–0.63 (Fig. 3b, Table S5). These changes in s.d. with increasing *n*_*f*_ also appear to be driven by greater variability when estimating leaf area centroids (Table S5). Similarly, asymptotic patterns of changes in trait hypervolumes with increasing sample size were observed at the individual farm scale. While there is a range of trait hypervolumes among farms for a given sampling intensity, within all sites trait hypervolume estimates began to converge where n~7–10 leaves per farm (Fig. S2, Tables S5, S6).

## Discussion

The development of standardized protocols for functional trait collection has contributed immensely to ensuring plant traits collected across studies, species, and ecosystems are comparable e.g.^29^. However, a definitive account of the sampling effort needed to derive accurate trait values, especially robust estimates of total ITV, has been more elusive e.g.^30^. Generally, more trait data will continuously change our understanding of what constitutes an accurate trait value for plant species or genotypes (Fig. 3a). Yet endlessly collecting samples for trait measurements is clearly impractical, and may not necessarily account for the major sources of trait variation within or across species e.g.^31^. Here we provide evidence that citizen-science efforts are a viable means to amass trait datasets, while also demonstrating that when designed carefully, such initiatives converge at robust trait values for (in our case) a given crop species (Fig. 3).

In our analysis here we asked, does the number of farms included in the sampling matter, and if so, how many farms is optimal? We show that leaf trait values and multi-dimensional hypervolume estimates saturate at a defined sampling intensity of sites (Fig. 3b). This saturation suggests that for a given crop under similar management, sampling geographically distinct sites is central in increasing the accuracy of trait values; however, at a certain point, adding new sites does not necessarily translate into markedly higher accuracy in trait values. Specifically, our analysis suggests that adding more than four farms did not drastically alter estimates of trait hypervolumes. In contrast, our data showed that setting a target number of observations (particularly if they are all derived from a single site) is a less effective approach to designing ITV sampling programs (Fig. 3a). Therefore, our data is broadly consistent with previous methodological assessments of how to most efficiently account for ITV in plants^30^, while simultaneously contributing to knowledge on the sampling effort required to capture trait expression within a crop. The applications of our findings are directly relevant for the successful deployment of farmer-based citizen science for the collection and consolidation of global crop trait data and the improvement of sampling protocols for scientist-led plant functional trait collections.

While trait database initiatives dedicated to wild plant species have been instrumental in enhancing our understanding of natural plant communities worldwide, there remains a lack of consolidated functional trait information for crops^5^. Certain techniques such as phylogenetically-based imputation methods^32^ have been proposed as a means to overcome “missing” trait data. However, research indicating that artificial selection drastically alters the trait syndromes of crops vs. their wild relatives indicates that such methods will likely result in inaccurate crop trait values^8^. Similarly, meta-analyses targeted at agronomic gray literature and databases have proved useful for amassing crop trait data in certain circumstances, but generally these approaches are ineffective for less common crops (i.e. anything other than rice, wheat, maize, or soy^15,16^). Ultimately, the lack of data derived from field-based collections remains a limitation to comprehensive, global-scale, and applied trait-based analyses in agroecosystems^5^.

We developed and tested farmer-led sampling programs as a viable means to amass crop trait data (Fig. 3). While comprehensive ecological analyses of our dataset are beyond the scope of our methodological research here, this type of crop trait data supports myriad novel hypotheses in both theoretical and applied plant science research. From a theoretical perspective, citizen-science-based data on crop traits (particularly ITV) could be employed in studies evaluating: (i) hypotheses on if or how artificial selection has led crops to deviate from “universal” plant trait spectra^9,13,33^; and (ii) the intentional and unintentional consequences of domestication through analyses of crop trait dimensionality^21,34^. From an applied perspective, such data has immediate implications for: (iii) refining process-based models of crop performance under environmental change and with agroecological practices^35^; (iv) better understanding genotype-by-environment responses of crops to environmental conditions^36^; and (v) informing farmer selection of crop genotypes in participatory plant breeding programs^37^.

Maximizing dominant agricultural production models may increase short-term yields in particular geographies and climatic zones, but at the expense of numerous global environmental problems that comprise the ecological foundations of food systems. Efforts to integrate ecological principles to achieve the optimal management of agroecosystems are critical to offset such production models. Recently, on-farm participatory variety evaluations using crowd-sourced citizen science have been shown to markedly increase the ability to select crops to adapt to climate change under heterogeneous environments^24^, while the use of crop functional traits has been shown to play a central role in farmer decision-making on sustainable management practices^38^. Merging the fields of agronomy and ecology with robust methodologies continues to enhance management prescriptions for low input place-based agriculture.

## Materials and Methods

### Experimental design and sample collection

The recent establishment of a national variety trialing program on organic farms across Canada provided a unique opportunity to tackle pressing questions regarding crop functional traits in agroecosystems. This network of organic farms participates in long-term vegetable crop variety trials. In the summer of 2018, we contacted 10 farms from four provinces in Canada to engage farmers in a leaf trait collection for five varieties of carrot (*Daucus carota* L.); all agreed (Fig. 1a). Research was carried out in accordance with guidelines outlined in an approved ethics protocol from the Behavioural Research Ethics Board, University of British Columbia, for research involving human participants. Informed consent was secured in advance of study participation. On each farm, farmers were instructed to isolate representative carrot plants that show no signs of disease or major pest damage with minimal plot edge effects. Leaf samples (*n* = 3) were collected six weeks post-emergence from all five varieties, resulting in *n* = 15 samples per farm and *n* = 150 individual observations in total. This collection represented a nearly 25-fold increase in trait data for this species compared to existing trait databases.

Farmers were instructed to pat leaf samples dry, and store flat in the labelled envelopes provided, each of which contained one small desiccant pack to maintain sample integrity during shipping (as leaves were shipped fresh, desiccant minimized the potential for mould growth and decomposition). Samples were then sent directly to the University of Toronto Scarborough, Canada (approximately two days in transit). Each leaf was immediately analyzed for eight morphological and chemical traits. During shipping 14 leaves were damaged, leaving a final dataset of *n* = 136 leaves. We measured leaf area (cm^2^) using a scanner and analyzed images using ImageJ software^39^. A low-force micrometer (No. 227–101, Mitutoyo Co., Mississauga, ON, Canada) was used to measure petiole diameter (mm) and a ruler to measure petiole length (cm), which are key traits related to harvestability^40^. Following these measurements, each leaf was dried at 60 °C to constant mass and weighed, and specific leaf area (SLA, cm^2^ g^−1^) was then calculated. Dried leaves were then ground into a fine powder using a ball mill (Retsch Ltd., Haan, Germany) and analyzed for total carbon (C) and nitrogen (N) concentrations (% mass basis) using a CN 628 elemental analyzer (LECO Instruments, Ontario, Canada).

### Statistical analyses – variation in traits across farms and varieties

To provide an initial assessment of trait variability across our sampling program, we calculated descriptive statistics for all individual traits across the entire dataset (*n* = 136). This was done by first assessing whether traits were normally or log-normally distributed, using a maximum-likelihood-based approach implemented with the ‘fitdistrplus’ R package^41^. Where traits were best described by a normal distribution (as per maximum likelihood scores), descriptive statistics were calculated means and standard deviations (SD); where traits were best described by log-normal distributions we calculated medians and median absolute deviation (MAD) values^14^. For all traits we also calculated coefficients of variation (CV) as an overall estimate of ITV.

Our next analysis was designed to assess if leaf traits varied as a function of variety, farm, and/or a variety-by-farm interaction term. This was done by first performing a principal components analysis (PCA) on all eight traits from our entire dataset (*n* = 136), and coupling this with a permutation multivariate analysis of variance (PerMANOVA). Both of these tests were implemented with the ‘vegan’ R package^42^, with the PerMANOVA being based on Euclidean distances and *n* = 10,000 permutations used. We also tested the statistical significance of individual PCA axes using the ‘testdim’ function in the ‘ade4’ R package^43^.

We then assessed how citizen science sampling intensity influenced estimates of *n*-dimensional plant trait hypervolumes, which describes a species niche space^44,45^. To do so we employed information on three individual traits including leaf area, leaf N, and SLA. Retaining these traits was done following recommendations by Blonder *et al*.^46^, who suggest hypervolumes should be estimated with the fewest dimensions possible, and for the following three primary reasons. First, all three of these traits factor prominently into “universal” functional trait spectra including the LES^47^ and the global spectrum of plant form and function^1^. Second, all three of these traits individually represent key inputs in the most widely employed models of crop yield and agroecosystem functioning (see review by^15^). And finally, these traits loaded strongly onto the first two PCA axes with leaf area being the trait associated most strongly with PCA axis 1 and leaf N/ SLA being the strongest associated with PCA axis 2 (Fig. 1b, Table S2).

Based on these three traits, hypervolume estimates were then calculated for the whole dataset (*H*_*obs*_, *n* = 136 leaves) using the ‘hypervolume_gaussian’ function in the ‘hypervolume’ R package^48^. All traits were log-transformed prior to analysis, and hypervolumes were estimated using a 0.05 quantile threshold such that each hypervolume contained 95% of the total probability distribution of *n* = 397 random samples per points. Kernel bandwidths for each trait were estimated independently using the ‘estimate_bandwidth’ function with values of 0.249, 0.105, and 0.071 for leaf area, SLA, and leaf N, respectively.

We then used a randomization procedure, broadly based on the approach taken by Lamanna *et al*.^49^, to evaluate how the accumulation of data points – either the number of leaves collected or the number of farms sampled – influenced estimates of 3-dimensional hypervolumes (refered to here as *H*_*rand*_ values). To do so, we generated datasets of *n* = 5, 10, 15,…, 135 randomly selected leaves through bootstrapping without replacement. At each sample size of leaves, 100 randomized datasets were generated and *H*_*rand*_ values were calculated using the exact methodologies described above (inclusive of the generation of new kernel density estimates for each trait dimension). We then derived a mean and standard deviation for *H*_*rand*_ values at each sample size for comparison to our *H*_*obs*_ estimate. Second, we generated randomized datasets that contained trait information from *n* = 1, 2, 3, …, 9 randomly selected farms within our dataset. For these analyses, the number of randomly constructed datasets at each farm sample size was also 100 (though note this procedure necessarily entailed bootstrapping with replacement where *n* ≤ 4 farms). Mean *H*_*rand*_ and standard deviations surrounding it were then calculated from all randomized dataset at a given number of farms.

Based on a preliminary visual assessment, we then chose to describe how *H*_*rand*_ changes as a function of the number of leaves and/or farms sampled by fitting non-linear models. Specifically, changes in *H*_*rand*_ at a given sample size were modeled as a function of the number of leaves randomly sampled (*n*_*l*_), following a negative exponential model fit of the form:

1 $${H}_{rand(l)}=a{({n}_{l})}^{-b}$$

where *H*_*rand(l)*_ represents the mean 3-dimensional trait hypervolume for a randomized dataset of a given sample size of leaves (*n*_*l*_), *a* represents the maximum mean *H* estimated for the lowest sample sizes (i.e. *n* = 5 leaves), and *b* represents the rate of reduction in *H*_*rand(l)*_ estimates as sample sizes increase.

Changes in *H*_*rand*_ in response to the number of farms sample (*n*_*f*_) was describe using an asymptotic model of the form:

2 $${H}_{rand(f)}=a+b\times \exp (\,-\,\exp (c)\times {n}_{f})$$

where *H*_*rand(f)*_ represents the mean 3-dimensional trait hypervolume for a randomized dataset of a given sample size of farms (*n*_*f*_), *a* represents the asymptote (i.e. maximum hypervolume), *b* represents the difference between the *y*-intercept and the asymptote, and *c* represents the log of the rate constant. Models and parameters in Eqs 1 and 2 were fit using maximum likelihood implemented in the ‘nls’ function in R. Finally, we used this analytical framework to evaluate how sampling intensity within individual farms influenced site-level estimates of trait hypervolumes. This entailed first performing the bootstrapping procedure to generate *n* = 100 datasets of *n* = 2, 3, …, 15 individual leaves, for each of the *n* = 10 individual farms separately. Equation 2 was then modified for each individual farm to assess how sampling intensity influences hypervolume estimates at the site-level, such that:

3 $${H}_{rand(lf)}=a+b\times \exp (\,-\,\exp (c)\times {n}_{lf})$$

where *a*, *b*, and *c* are as in Eq. 2, and *H*_*rand(lf)*_ represents the mean 3-dimensional trait hypervolume for a randomized dataset for a given sample size of leaves within a given farm (*nl*_*f*_).

## Supplementary information

Supplementary Information_Revised
