# Supplementary material for: Accumulating crop functional trait data with citizen science
Source: Sci Rep. 2019 Oct 31;9:15715. doi: 10.1038/s41598-019-51927-x (PMC6823441; doi:10.1038/s41598-019-51927-x)
Supplement: Supplementary file 1 — Supplementary Information_Revised [file 41598_2019_51927_MOESM1_ESM.pdf]

Supplementary Information for: Accumulating crop functional trait data with citizen science

Marney E. Isaac[1, 2, *] and Adam R. Martin[1]

[1] Department of Physical and Environmental Sciences, University of Toronto Scarborough, Toronto, Canada.
[2] The Centre for Critical Development Studies, University of Toronto Scarborough, Toronto, Canada.
[*]Corresponding author contact: Marney.isaac@utoronto.ca

Table S1. Descriptive statistics for eight leaf traits measured across *n*=136 carrot leaves collected through a citizen-science trait collection initiative. Distributions were determined based on best maximum-likelihood scores (highlighted in bold). Where traits were best described by a normal distribution descriptives were calculated as means and standard deviations (SD), whereas descriptives for log-normally distributed traits were calculated as medians and median absolute deviations (MAD). Coefficients of variation (CV) are presented for all traits.

Trait	Normal	log-Normal	Range	Mean/median	SD/MAD	CV
Petiole diameter	-182.2	**-180.5**	0.60-5.76	2.6	0.8	34.2
Petiole length	**-494.4**	-494.9	14-58	32.5	9.2	28.4
Leaf mass	**43.0**	71.9	0.02-1.13	0.27	0.18	65.6
Leaf area	-649.4	**-632.5**	3.5-193.0	44.4	24.9	59.7
SLA	-726.5	**-719.6**	83.7-403.8	183.5	45.2	27.0
Leaf C	**-269.1**	-270.6	35.0-46.3	40.8	1.8	4.3
Leaf N	**-152.9**	-155.9	2.6-5.9	4.3	0.8	17.3
Leaf C:N	-253.2	**-247.7**	7.1-14.4	9.8	1.6	15.7

Table S2. Principal component axis loadings for eight leaf traits measured across *n*=136 carrot leaves collected through a citizen-science trait collection initiative. Visual representation of PCA axis 1 and 2 loadings are presented in Figure 1B in the main text.

Trait	PC1	PC2	PC3	PC4	PC5	PC6
Petiole diameter	-1.15343	1.0837	0.60541	0.52753	-0.4262	0.88104
Petiole length	-0.78152	1.1237	1.22784	0.20581	-0.2805	-0.77259
Leaf mass	-1.73694	0.4935	-0.83087	-0.08955	0.2792	-0.16986
Leaf area	-1.78463	0.12	-0.59084	0.25465	0.6724	-0.08523
SLA	0.09911	-1.1465	0.81269	0.96876	1.085	0.05564
Leaf C	-0.92244	-0.4859	0.89799	-1.39495	0.4457	0.18821
Leaf N	-0.86655	-1.6709	0.1266	-0.1775	-0.5131	0.05051
Leaf C:N	1.008	1.4056	0.01764	-0.46464	0.8264	0.17574

Table S3. Results of a permutational multivariate analysis of variance (PerMANOVA) predicting multivariate leaf trait syndromes in *n*=136 carrot leaves as a function of variety identify, farm, and a variety-by-farm interaction term. The PerMANOVA results here were based on *n*=10,000 permutations.

**Factor**	**D.f.**	**Sums Of Sqs.**	**Mean Sqs.**	***F***	r^2	***p***
**Farm**	**9**	**139363**	**15484.8**	**5.5253**	**0.29534**	**0.001**
Variety	4	15516	3879	1.3841	0.03288	0.196
Farm-by-variety	32	64768	2024	0.7222	0.13726	0.924
Residuals	90	252226	2802.5		0.53452	
Total	135	471874			1	

Table S4. Randomization analysis assessing trait hypervolumes and estimated centroids of three leaf traits as a function of sampling intensity (measured as the number of leaf observations) in *Daucus carota.* All estimates are based on *n*=100 randomized datasets at a given sample size, generated through bootstrapping without replacement. Values for the entire dataset are where *n* observations=136, therefore no standard deviations are provided for at that sample size.

	**Hypervolume**		**Leaf area**		**SLA**		**Leaf N**	
***n* Observations**	**Mean**	**SD**	**Mean**	**SD**	**Mean**	**SD**	**Mean**	**SD**
136	2.36	–	3.57	–	5.19	–	1.42	–
135	2.02	0.21	3.57	0.07	5.18	0.03	1.41	0.02
130	2.09	0.19	3.58	0.06	5.18	0.03	1.42	0.02
125	2.09	0.28	3.57	0.08	5.18	0.03	1.42	0.02
120	2.08	0.22	3.57	0.08	5.18	0.03	1.41	0.02
115	1.95	0.16	3.62	0.08	5.20	0.03	1.42	0.02
110	2.11	0.19	3.60	0.08	5.18	0.04	1.41	0.01
105	2.13	0.21	3.56	0.08	5.18	0.04	1.42	0.02
100	2.17	0.23	3.57	0.05	5.20	0.03	1.42	0.02
95	2.04	0.23	3.61	0.04	5.19	0.05	1.42	0.02
90	2.14	0.31	3.58	0.04	5.18	0.03	1.42	0.02
85	2.07	0.22	3.60	0.06	5.18	0.05	1.41	0.02
80	2.03	0.25	3.59	0.07	5.19	0.04	1.42	0.03
75	2.15	0.44	3.61	0.07	5.20	0.04	1.42	0.02
70	2.26	0.50	3.57	0.08	5.17	0.04	1.43	0.02
65	2.27	0.27	3.60	0.10	5.17	0.02	1.42	0.02
60	2.22	0.52	3.56	0.10	5.18	0.05	1.43	0.02
55	2.14	0.35	3.60	0.11	5.19	0.05	1.44	0.04
50	2.38	0.59	3.58	0.08	5.18	0.05	1.42	0.03
45	2.20	0.43	3.55	0.13	5.18	0.05	1.42	0.03
40	2.28	0.62	3.57	0.11	5.21	0.04	1.43	0.03
35	2.15	0.65	3.59	0.18	5.20	0.05	1.43	0.02
30	2.35	0.65	3.66	0.11	5.22	0.05	1.44	0.04
25	2.30	0.87	3.66	0.19	5.19	0.04	1.44	0.04
20	2.20	0.46	3.65	0.19	5.15	0.08	1.41	0.04
15	2.74	1.16	3.68	0.20	5.20	0.08	1.42	0.06
10	2.74	0.90	3.50	0.27	5.21	0.10	1.47	0.06
5	2.64	3.09	3.71	0.37	5.17	0.09	1.44	0.07

Table S5. Randomization analysis assessing trait hypervolumes and estimated centroids of three leaf traits as a function of sampling intensity (measured as the number of farms sampled) in *Daucus carota.* All estimates are based on *n*=100 randomized datasets at a given sample size, generated through bootstrapping without replacement. Values for the entire dataset are where *n* observations=10, therefore no standard deviations are provided for at that sample size.

	Hypervolume		Leaf area		SLA		Leaf N	
***n* Farms**	**Mean**	**SD**	**Mean**	**SD**	**Mean**	**SD**	**Mean**	**SD**
10	2.36	–	3.57	–	5.19	–	1.42	–
9	2.28	0.15	3.55	0.08	5.19	0.02	1.42	0.02
8	2.26	0.30	3.60	0.10	5.18	0.03	1.42	0.02
7	2.33	0.29	3.57	0.10	5.18	0.03	1.42	0.03
6	2.29	0.56	3.63	0.17	5.20	0.04	1.44	0.02
5	2.08	0.47	3.65	0.21	5.17	0.07	1.40	0.04
4	1.96	0.51	3.55	0.25	5.18	0.09	1.42	0.07
3	1.70	0.51	3.70	0.35	5.17	0.09	1.41	0.07
2	1.39	0.63	3.74	0.29	5.25	0.09	1.45	0.10
1	0.83	0.39	3.72	0.47	5.19	0.17	1.46	0.15

Table S6. Randomization analysis assessing trait hypervolumes and estimated centroids of three leaf traits as a function of sampling intensity (measured as the number of farms sampled) in *Daucus carota*, within 10 individual farms. All estimates are based on *n*=100 randomized datasets at a given sample size generated through bootstrapping without replacement. Values for the entire datasets are where *n* observations=15, therefore no standard deviations are provided for at that sample size. Farm numbers here are consistent with those in Figures 1, S1, and S2.

**Farm**	***n* observations**	**Hypervolume**		**Leaf area**		**SLA**		**Leaf N**	
		**Mean**	**SD**	**Mean**	**SD**	**Mean**	**SD**	**Mean**	**SD**
	2	1.86	1.95	2.98	0.31	5.19	0.16	1.4	0.08
1	3	0.96	0.66	3.09	0.22	5.2	0.09	1.45	0.07
	4	1.7	1.33	3.18	0.25	5.11	0.13	1.42	0.11
	5	2.09	0.55	3.05	0.2	5.08	0.04	1.41	0.06
	6	2.39	0.8	3.11	0.17	5.1	0.07	1.41	0.04
	7	1.82	0.5	3.01	0.15	5.1	0.07	1.4	0.04
	8	1.84	0.4	3.02	0.13	5.08	0.04	1.38	0.03
	9	1.72	0.53	3.1	0.11	5.12	0.05	1.41	0.04
	10	1.87	0.43	3.1	0.14	5.1	0.05	1.39	0.03
	11	1.86	0.25	3.07	0.09	5.1	0.04	1.4	0.02
	12	1.83	0.23	3.02	0.07	5.09	0.02	1.39	0.02
	13	1.79	0.2	3.08	0.05	5.1	0.03	1.4	0.01
	14	1.83	0.03	3.08	0.01	5.09	0.01	1.4	0
	15	1.77	–	3.06	–	5.06	–	1.4	–
	2	0.52	1.17	3.89	0.24	5.3	0.06	1.46	0.11
2	3	0.6	0.42	3.76	0.29	5.25	0.13	1.49	0.05
	4	0.79	0.65	3.84	0.18	5.29	0.08	1.47	0.05
	5	0.76	0.51	3.83	0.06	5.27	0.08	1.46	0.05
	6	0.81	0.31	3.83	0.11	5.28	0.05	1.47	0.03
	7	0.93	0.29	3.83	0.11	5.32	0.04	1.44	0.03
	8	0.96	0.29	3.79	0.06	5.31	0.04	1.45	0.04
	9	0.92	0.26	3.83	0.07	5.3	0.04	1.45	0.02
	10	0.87	0.21	3.8	0.07	5.31	0.02	1.46	0.02
	11	0.84	0.12	3.8	0.05	5.3	0.02	1.45	0.02
	12	0.86	0.01	3.79	0.01	5.31	0.01	1.45	0
	13	0.86	0.01	3.79	0.01	5.3	0	1.45	0
	14	0.87	0.02	3.79	0.01	5.31	0.01	1.45	0
	15	0.87	–	3.8	–	5.3	–	1.45	–
	2	0.57	0.82	3.71	0.16	5.33	0.17	1.62	0.04
3	3	0.26	0.18	3.79	0.17	5.28	0.16	1.63	0.05

	4	0.71	0.5	3.74	0.17	5.42	0.12	1.64	0.03
	5	0.54	0.39	3.8	0.15	5.37	0.14	1.63	0.03
	6	0.61	0.28	3.87	0.09	5.35	0.12	1.64	0.02
	7	0.78	0.35	3.76	0.08	5.35	0.11	1.63	0.02
	8	0.62	0.2	3.78	0.09	5.35	0.06	1.63	0.01
	9	0.58	0.23	3.8	0.07	5.39	0.08	1.65	0.01
	10	0.62	0.12	3.78	0.06	5.39	0.06	1.64	0.01
	11	0.62	0.13	3.79	0.05	5.38	0.06	1.64	0.01
	12	0.55	0.1	3.8	0.04	5.35	0.04	1.64	0.01
	13	0.58	0.08	3.76	0.02	5.38	0.03	1.63	0.01
	14	0.56	0.06	3.78	0.03	5.38	0.02	1.64	0.01
	15	0.55	–	3.78	–	5.38	–	1.64	–
4	2	0.45	0.88	3.75	0.23	5.24	0.13	1.27	0.06
	3	0.54	0.5	3.74	0.24	5.2	0.08	1.29	0.06
	4	0.76	0.36	3.83	0.17	5.27	0.07	1.29	0.04
	5	0.88	0.42	3.81	0.16	5.25	0.05	1.31	0.03
	6	0.64	0.25	3.78	0.16	5.25	0.04	1.28	0.04
	7	0.74	0.15	3.87	0.12	5.27	0.05	1.29	0.02
	8	0.72	0.12	3.88	0.09	5.26	0.03	1.3	0.02
	9	0.71	0.17	3.82	0.05	5.24	0.04	1.29	0.02
	10	0.68	0.18	3.82	0.06	5.26	0.02	1.28	0.03
	11	0.69	0.08	3.83	0.04	5.26	0.03	1.29	0.02
	12	0.68	0.06	3.83	0.03	5.25	0.02	1.29	0.02
	13	0.7	0.08	3.81	0.04	5.26	0.01	1.29	0.01
	14	0.7	0.04	3.81	0.01	5.25	0.01	1.29	0.01
	15	0.67	–	3.81	–	5.25	–	1.28	–
5	2	0.34	0.35	4.38	0.32	5.19	0.11	1.58	0.03
	3	0.36	0.34	4.21	0.26	5.27	0.13	1.57	0.03
	4	0.48	0.46	4.39	0.27	5.21	0.1	1.57	0.02
	5	0.53	0.38	4.43	0.17	5.2	0.11	1.57	0.02
	6	0.47	0.27	4.31	0.16	5.27	0.11	1.56	0.01
	7	0.46	0.25	4.35	0.18	5.26	0.09	1.56	0.01
	8	0.41	0.18	4.4	0.15	5.24	0.08	1.57	0.01
	9	0.52	0.17	4.4	0.11	5.23	0.06	1.57	0.01
	10	0.5	0.19	4.38	0.11	5.27	0.06	1.56	0.02
	11	0.53	0.14	4.4	0.06	5.26	0.03	1.57	0.01
	12	0.51	0.12	4.37	0.09	5.26	0.06	1.57	0.01
	13	0.52	0.08	4.39	0.06	5.26	0.04	1.57	0
	14	0.53	0.06	4.41	0.04	5.26	0.02	1.57	0
	15	0.51	–	4.41	–	5.26	–	1.57	–
6	2	0.17	0.24	3.99	0.25	5.44	0.11	1.44	0.06

	3	0.27	0.17	4	0.2	5.39	0.08	1.44	0.05
	4	0.28	0.17	3.96	0.17	5.43	0.06	1.43	0.02
	5	0.36	0.15	3.99	0.07	5.37	0.08	1.44	0.03
	6	0.36	0.15	4.06	0.11	5.41	0.06	1.43	0.02
	7	0.39	0.11	4.07	0.07	5.37	0.04	1.43	0.01
	8	0.35	0.12	4.05	0.08	5.42	0.05	1.45	0.02
	9	0.41	0.09	4.04	0.05	5.4	0.02	1.44	0.02
	10	0.36	0.09	4.05	0.07	5.41	0.04	1.45	0.01
	11	0.36	0.09	4.04	0.04	5.41	0.04	1.44	0.01
	12	0.38	0.07	4.03	0.04	5.41	0.02	1.44	0.01
	13	0.38	0.04	4.03	0.02	5.41	0.02	1.45	0.01
	14	0.39	0.02	4.04	0.02	5.4	0.01	1.44	0.01
	15	0.39	—	4.04	—	5.4	—	1.44	—
7	2	0.72	1.09	3.95	0.14	5.07	0.14	1.22	0.1
	3	0.6	0.66	3.96	0.14	5.06	0.11	1.21	0.1
	4	0.84	0.51	3.92	0.14	5.07	0.09	1.22	0.08
	5	0.75	0.37	3.88	0.1	5.07	0.08	1.17	0.06
	6	0.93	0.2	3.92	0.07	5.06	0.03	1.19	0.03
	7	0.68	0.42	3.91	0.11	5.08	0.1	1.21	0.06
	8	0.76	0.28	3.9	0.04	5.06	0.04	1.2	0.03
	9	0.75	0.17	3.89	0.08	5.08	0.05	1.2	0.05
	10	0.76	0.21	3.92	0.06	5.09	0.03	1.22	0.03
	11	0.77	0.08	3.88	0.07	5.07	0.03	1.17	0.03
	12	0.77	0.14	3.9	0.06	5.08	0.02	1.2	0.03
	13	0.77	0.19	3.88	0.03	5.07	0.04	1.19	0.02
	14	0.78	0.14	3.89	0.06	5.08	0.01	1.2	0.04
	15	0.78	—	3.9	—	5.07	—	1.2	—
8	2	0.61	0.75	4.12	0.17	5.01	0.14	1.44	0.1
	3	0.45	0.38	4.08	0.26	5.02	0.12	1.44	0.1
	4	0.67	0.29	4.2	0.12	5.06	0.08	1.42	0.06
	5	0.62	0.29	4.07	0.13	4.98	0.08	1.42	0.06
	6	0.61	0.19	4.09	0.09	5.03	0.08	1.43	0.04
	7	0.76	0.21	4.16	0.07	5.02	0.04	1.41	0.03
	8	0.74	0.16	4.13	0.08	5	0.04	1.41	0.03
	9	0.74	0.2	4.12	0.04	5.04	0.04	1.42	0.04
	10	0.73	0.1	4.14	0.06	5.01	0.05	1.41	0.01
	11	0.86	0.11	4.11	0.06	5.02	0.01	1.41	0.02
	12	0.73	0.1	4.14	0.06	5.02	0.03	1.42	0.01
	13	0.77	0.07	4.14	0.03	5.03	0.02	1.41	0.01
	14	0.81	0.04	4.16	0.03	5.02	0.01	1.41	0
	15	0.77	—	4.14	—	5.02	—	1.41	—

9	2	1.66	2.08	2.77	0.47	4.98	0.24	1.25	0.04
	3	1.02	0.73	3.05	0.09	4.86	0.11	1.22	0.04
	4	1.58	1.21	2.72	0.25	5.02	0.17	1.25	0.04
	5	2.03	1.55	2.82	0.2	4.94	0.14	1.22	0.03
	6	1.77	0.74	2.7	0.24	4.93	0.13	1.23	0.05
	7	1.64	0.63	2.87	0.24	4.99	0.11	1.22	0.04
	8	2	1.01	2.8	0.17	4.92	0.08	1.24	0.04
	9	1.81	0.87	2.82	0.13	4.95	0.06	1.23	0.02
	10	1.81	0.47	2.75	0.13	4.95	0.07	1.24	0.03
	11	1.81	0.43	2.73	0.09	4.95	0.08	1.24	0.02
	12	1.59	0.45	2.81	0.14	4.97	0.07	1.24	0.02
	13	1.93	0.26	2.76	0.04	4.96	0.03	1.24	0.01
	14	1.7	0.19	2.76	0.05	4.96	0.02	1.25	0.02
	15	1.81	—	2.78	—	4.96	—	1.24	—
10	2	0.73	0.77	3.44	0.14	5.2	0.13	1.65	0.05
	3	0.48	0.4	3.32	0.28	5.21	0.15	1.67	0.05
	4	0.71	0.71	3.32	0.2	5.29	0.08	1.67	0.04
	5	0.93	0.45	3.2	0.27	5.26	0.09	1.66	0.02
	6	0.6	0.44	3.32	0.16	5.27	0.09	1.67	0.02
	7	0.66	0.47	3.2	0.14	5.27	0.1	1.67	0.02
	8	0.66	0.28	3.35	0.17	5.24	0.1	1.66	0.02
	9	0.87	0.26	3.22	0.13	5.26	0.05	1.66	0.02
	10	0.78	0.24	3.22	0.11	5.28	0.04	1.67	0.01
	11	0.77	0.26	3.31	0.11	5.25	0.03	1.66	0.01
	12	0.74	0.19	3.3	0.11	5.27	0.04	1.66	0.01
	13	0.78	0.12	3.25	0.07	5.27	0.02	1.67	0.01
	14	0.82	0.02	3.25	0.02	5.28	0.01	1.66	0
	15	0.71	—	3.27	—	5.27	—	1.67	—

Table S7. Parameters and diagnostics for asymptotic models predicting mean trait hypervolumes as a function of number of leaves sampled, within 10 individual farms. Model parameters (and associated standard errors (SE)) were fit following Equation 3 in the main text for each individual farm (where Farm Identity corresponds to Table S6, and Figures 1, S1, and S2).

	**Farm Identity**									
**Model parameters**	**1**	**2**	**3**	**4**	**5**	**6**	**7**	**8**	**9**	**10**
a	1.87	0.88	0.61	0.71	0.51	0.38	0.78	1.91	1.83	1.83
a SE	0.15	0.02	0.05	0.02	0.02	0.01	0.03	15.02	0.17	0.17
B	-1.06	-0.34	-0.8	-0.24	-0.6	-0.22	-1.26	-0.27	-0.93	-0.93
b SE	1.21	0.1	1	0.2	0.23	0.06	1.19	2.42	0.6	0.6
c	0.71	1.24	0.87	1.94	0.86	1.45	0.73	0.26	0.64	0.64
c SE	1.24	0.26	1.31	0.96	0.38	0.22	1.08	0.61	0.72	0.72
Model AIC	11.86	-40.27	-15.37	-29.92	-45.01	-67.51	-27.87	-31.22	3.76	3.76

61

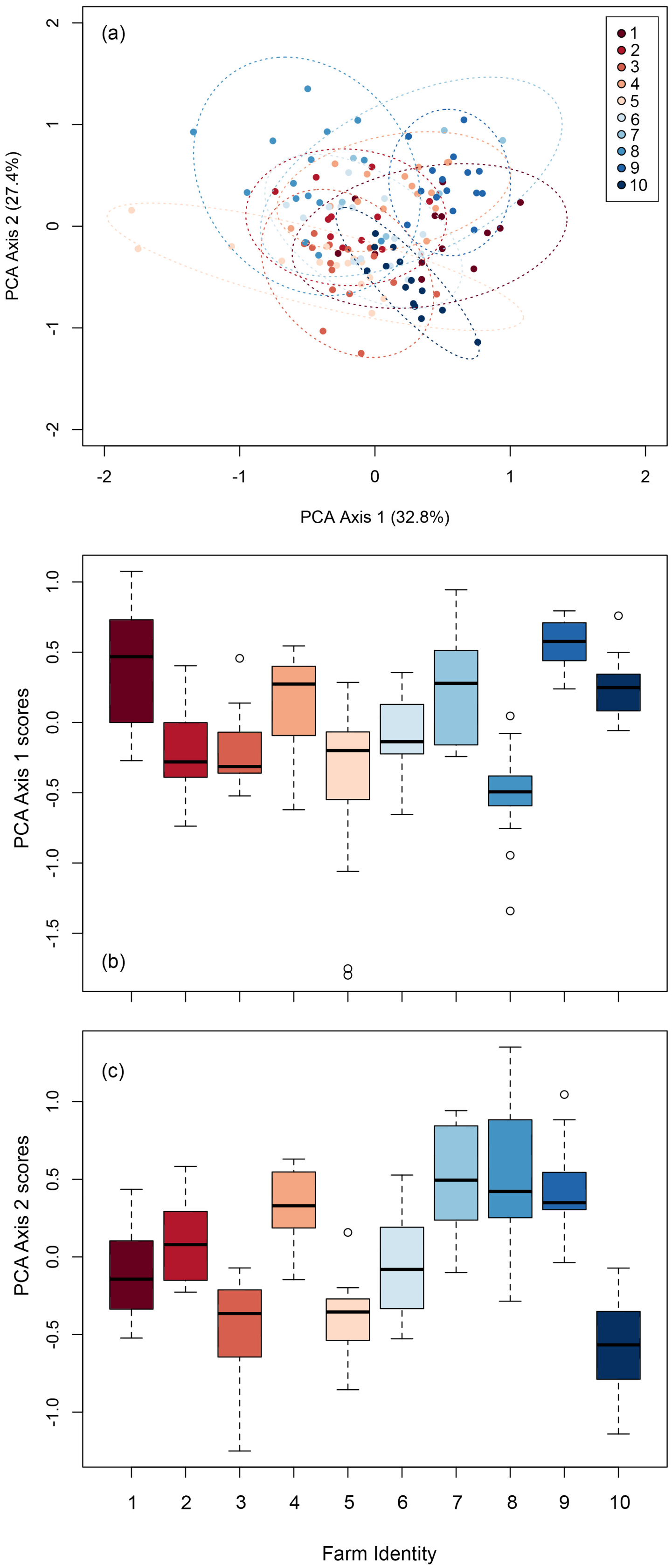
(a)
PCA Axis 1 (32.8%)
PCA Axis 2 (27.4%)
1
2
3
4
5
6
7
8
9
10
(b)
PCA Axis 1 scores
(c)
PCA Axis 2 scores
Farm Identity

Figure S1. Differences in multivariate trait syndromes of *Daucus carota* across10 farms used in a citizen science trait collection initiative. Panel A represents the same principal components analysis (PCA) evaluating multivariate differences in traits that is presented in Figure 1b in the main text. However in Panel A here, confidence ellipses are shown that represent 95% confidence limits surrounding multivariate trait syndromes for each individual farm; PCA vectors have been removed for clarity but are presented in Figure 1b. Panels B and C present boxplots of PCA axis 1 and 2 scores, respectively, for each individual farm. Corresponding farm locations are available in Figure 1a of the main text.

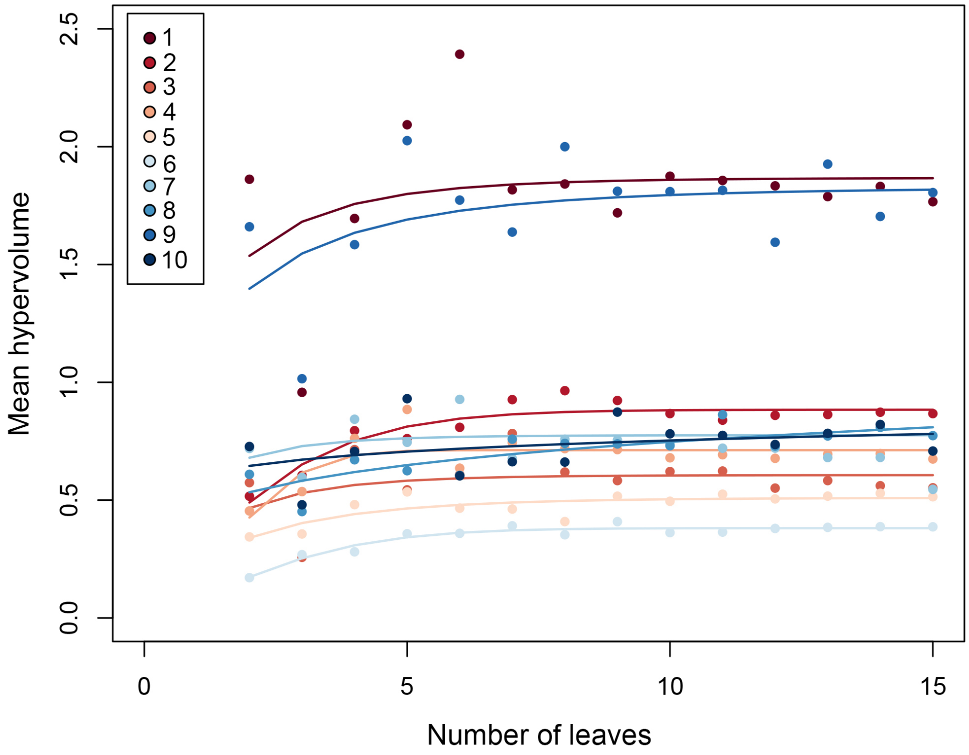

Figure S2. Changes in estimated leaf trait hypervolumes as a function of sample size, within 10 individual farms. Trend lines correspond to asymptotic models fit to each farm individually, with model parameters and diagnostics presented in Table S7. For clarity, only the mean hypervolume estimated for each farm-by-number of leaves combination (generated through bootstrapping and Equation 3 as described in the main text) are presented here; standard deviations associated with each data point are presented in Table S6.
